# Developmental and Age-Related RAB-3 phenotypes in a *C. elegans* Tauopathy Model

**DOI:** 10.17912/micropub.biology.002113

**Published:** 2026-05-29

**Authors:** Aidan Anderson, Karen Kim Guisbert, Melissa Borgen

**Affiliations:** 1 Biomedical Engineering and Science, Florida Institute of Technology, Melbourne, FL, US; 2 Department of Biology, University of Nebraska at Omaha, Omaha, NE, US

## Abstract

Tauopathies are neurodegenerative diseases characterized by aggregation of Tau into neurofibrillary tangles. Elucidation of cellular phenomena occurring in degenerative states will be important for long-term mitigation strategies. Here we show two phenotypes involving the synaptic vesicle protein RAB-3 in a *Caenorhabditis elegans* TauV337M model: a developmental reduction of RAB-3 and an age-related mislocalization in motor axons. We further show that the developmental reduction of RAB-3 is not due to changes in transcriptional expression. We suggest axonal transport and/or synaptic vesicle recycling defects are responsible for developmental and age-related phenotypes.

**Figure 1. RAB-3 levels are broadly decreased in development and aberrantly distributed in aged TauTg animals:** A) Diagram of GABAergic motor cord in *C. elegans* . B) Confocal images of the RAB-3 ::GFP in the nerve cord at L4 in the TauTg genotype ( *bkIs10* ) and N2 age-matched control ( *jsIs682* [ rab-3 p::GFP:: rab-3 + lin-15 (+)]). White boxes show magnified 100µm ROI of dorsal nerve cord. Arrow heads note regions of non-uniform distribution. Scale bar = 100µm. C) Percent of animals that exhibited non-uniform RAB-3 distribution at L4 and day 6 of adulthood (D6), * p < 0.05, ** p<0.01. D) Average motor cord RAB-3 ::GFP fluorescence intensity at L4, day 2 (D2) and D6 of adulthood, * p<0.05 , ** p<0.01, *** p < 0.001.). E) RAB-3 ::GFP puncta density of dorsal nerve cord (puncta per 100µm),*** p<0.001 F) Average motor cord RAB-3 ::GFP fluorescence area at L4, D2 and D6, * p<0.05 , ** p<0.01, *** p < 0.001). G) Confocal images of L4 and day 1 worms expressing GFP in the GABAergic motor cord. L4 WT (top) shows no breaks in the motor cord. L4 TauTg shows a single small break, noted by an arrowhead. D1 TauTg shows two moderately sized breaks. Scale bar = 100µm. H) Average number of motor cord breaks at L4, D1 and D6, ** p<0.01 ***p<0.001, ****p<0.0001. I) Relative expression of *rab-3* mRNA compared to 2 housekeeping genes ( *act-1* , *tba-2* ). J) Relative expression of *aex-3* mRNA compared to 2 housekeeping genes ( *act-1* , *tba-2* ). K) Percent non-uniform distribution of SNB-1 ::GFP at L4 and D6, ns = not significant. L) Average intensity of SNB-1 GFP at L4, D2, and D6. M) SNB-1 ::GFP puncta density in dorsal nerve cord (puncta per 100µm) at L4, D2, and D6. N) Average SNB-1 ::GFP fluorescence area at L4, D2, and D6. O) Top: D6 WT RAB-3 ::GFP puncta distribution with mCherry cell fill; bottom: dual channel intensity plot of RAB-3 ::GFP and mCherry, representing area highlighted by white box in image. P) Top: D6 TauTg RAB-3 ::GFP puncta distribution with mCherry cell fill; bottom: dual channel intensity plot of RAB-3 ::GFP and mCherry, representing area highlighted by white box in image. This shows an area of non-uniform RAB-3 ::GFP distribution in areas where axons are intact (Red asterisk).

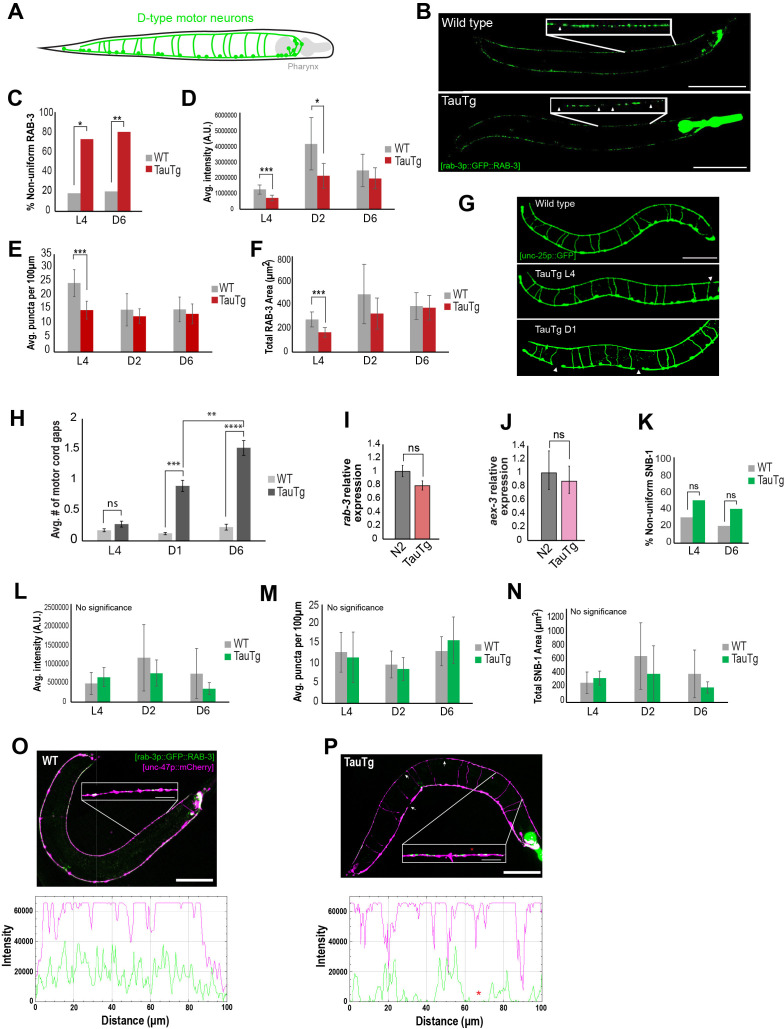



## Description

Tauopathies are neurodegenerative diseases characterized by aggregation of Tau protein into neurofibrillary tangles, contributing to loss of neuronal function. Previous work has shown that expression of human Tau containing the FTD17P-assocaiated V337M allele (TauTg) causes progressive degeneration of the *C. elegans* motor cord (Kraemer BC et al., 2003). Synapse loss is a hallmark of neurodegenerative diseases and is observed prior to neuron death (Selkoe DJ, 2002;Wilson DM, 3rd et al., 2023). Understanding synaptic changes preceding axon degeneration and neuron death are critical and remain unclear in the TauTg model. Here, we show that the TauTg model exhibits both developmental and age-related RAB-3 phenotypes in the *C. elegans* GABAergic motor cord.

The motor cord spans the dorsal and ventral lengths of the worm, with characteristic synaptic patterns for SNB-1 /synaptobrevin (Balklava Z et al., 2015;Grill B et al., 2007;Hallam SJ and Jin Y, 1998;Mahoney TR et al., 2006) ( Figure 1A, B). We crossed strains containing transgenes expressing GFP-tagged RAB-3 ( *jsIs682* ) or GFP-tagged SNB-1 ( *juIs1* ) to the TauTg model to visualize presynapses in the disease model, as well as wild type (WT) sibling controls for comparison. Similar to previous studies, in WT animals, we show a uniform distribution of RAB-3 puncta along the dorsal nerve cord. However, RAB-3 distribution is non-uniform in 73% of TauTg animals, with distinct gaps between puncta (Fig 1B,C). The percentage of animals with this aberrant distribution slightly increases with age, as 80% animals showed RAB-3 distribution phenotypes at day 6 of adulthood. While the percentage of animals affected doesn't increase significantly, we observed more severe mis-localization with age, with larger stretches of axons without RAB-3 puncta (not shown). This may indicate synaptogenesis or transport defects on day 1 of adulthood.&nbsp; We suspect increasing mis-localization as disease progresses resulting from disrupted RAB-3 transport or trafficking during synaptic vesicle cycling.

We then tested another synaptic marker, SNB-1 ::GFP, to see if there is a broad synaptic defect, or if the defects are specific to RAB-3 . SNB-1 is uniformly distributed in WT animals shown here as 30% non-uniform distribution at L4 and 20% at day 6 indicating no are-related deficits (Fig 1K). Comparatively, TauTg showed more animals with non-uniform distribution at L4, but there was no significant difference compared to WT. Interestingly, at day 6, there is still no significant difference in SNB-1 distribution between TauTg and WT. Taken together, there is a RAB-3 specific phenotype and not a broad disruption of synaptic machinery.

It is important to note that while axon degeneration occurs in the TauTg model, degeneration occurs as small gaps in the motor cord (Fig 1G arrowhead), leaving the motor cord axons still largely intact. Most animals at the L4 stage don't exhibit these degenerative gaps in the motor cord. However, the number of gaps increase with age (Fig 1H). Despite this, gaps are small and do not span the entire motor cord (Fig 1G). Thus, RAB-3 non-uniformity is not due to missing axons in those locations. To confirm, we assessed RAB-3 ::GFP uniformity in a GABAergic motor neuron mCherry cell fill ( *unc-47p::mCherry* ), which shows that non-uniformity occurs in axon regions that have not degenerated (Fig 1O, P).

We measured RAB-3 with multiple metrics at different points across lifespan, including puncta density, total RAB-3 area, and intensity (Fig 1D-F). Across all measures, RAB-3 is significantly lower in the TauTg animals compared to WT at the L4 larval stage. While intensity stays lower at day 2 of adulthood, TauTg animals generally “catch up” to WT RAB-3 levels. It is worth noting that WT levels of RAB-3 intensity drop between day 2 and day 6, showing an age-related decline, with a similar trend for total RAB-3 area. RAB-3 intensity in TauTg animals is sharply reduced at day 2 of adulthood compared to age-matched WT, with day 2 TauTg intensity similar to levels associated with advanced age in WT (day 6).&nbsp; In contrast to RAB-3 , there is no significant change to SNB-1 levels in TauTg by any metric: intensity, area, or density ( Fig. 1L- N), reinforcing that there is a RAB-3 specific effect in the TauTg animals.

As we observed lower RAB-3 levels in L4 TauTg, we tested whether this is due to changes in *rab-3* mRNA expression. We performed RT-qPCR, comparing *rab-3* mRNA levels to 2 different housekeeping genes: *tba-1* and *act-1* . We found no significant difference in *rab-3* levels in TauTg animals ( Fig. 1I ). It is noteworthy that the promoter driving expression of the humanized TauTg construct is *aex-3 p.* AEX-3 is a RAB-3 guanine exchange factor and as such has a regulatory interactions with RAB-3 during synaptic transmission, development, and vesicle recycling (Bae H et al., 2016;Bhat JM and Hutter H, 2016;Iwasaki K et al., 1997). If expression of TauTg causes promoter squelching, *aex-3* expression may be reduced, which could reduce RAB-3 localization to synaptic vesicles. Thus, we measured *aex-3* mRNA levels in TauTg animals compared to WT sibling controls. Results indicate no change in *aex-3* expression (Fig 1J). Taken together, we show that reduction in developmental RAB-3 is not occurring at the transcriptional level.

In summary, our data show 1) developmental delay in RAB-3 protein expression; 2) mis-localization of axonal RAB-3 ; 3) RAB-3 , but not SNB-1 , is specifically affected by TauTg, and 4) there is no broad loss of synaptic vesicles prior to axon degeneration. Our results reveal a progressive loss of RAB-3 in axonal vesicles, but not an overall drop in RAB-3 levels. There are many studies that show RAB-3 /Rab3 expression differs in neurodegenerative diseases, but they show changes in later stages, not during development (He H et al., 2025). Changes in Rab3 expression in neurodegenerative disease is well-documented, though these changes vary by cell type, brain region, and disease. The mechanisms underlying RAB-3 mis-localization are unclear, though we suspect transport of RAB-3 from the soma to axons is progressively disrupted. The RAB-3 specific phenotype could also be affected by defects in endosomal sorting of RAB-3 after synaptic vesicle endocytosis in axons. We see no localization defects with SNB-1 , which stays embedded in recycled membrane. RAB-3 is dissociated after endocytosis and GTP hydrolysis, suggesting that localization of RabGEF, AEX-3 , could also be disrupted in TauTg (Pavlos NJ and Jahn R, 2011). Additionally, local translation of *rab-3* could be affected. Further work is needed to uncover molecular mechanisms.&nbsp;

Several studies have explored links between Autism Spectrum Disorder (ASD) and Alzheimer's Disease (Hu W et al., 2023;Rhodus EK et al., 2022), including studies showing reduced GABAergic synthesis and reduction of GABAergic cells in ASD (Ariza J et al., 2018;Hashemi E et al., 2018;Hong T et al., 2020). However, the studies focus more on APP than Tau and they do not describe specific effects on Rab3.&nbsp; We show a novel developmental delay in RAB-3 expression in the TauTg model, which is of interest as Tau and its interactors have also been implicated in developmental disorders, such as ASD (Tai C et al., 2020;Villavicencio-Tejo F et al., 2025), Dravet syndrome (Gheyara AL et al., 2014) , and microcephaly (Sokol DK and Lahiri DK, 2023).

## Methods

***C. elegans* microscopy and image analysis**

*C. elegans* strains were bleach synchronized and maintained on standard NGM growth media seeded with OP50 *E. coli.* Worms were cultured until desired growth stage (L1-D6) at 22°C as described (Brenner 1974). Worms were mounted on standard microscope slides with a 2% agarose pad and paralyzed with 5mM levamisole for imaging. Confocal imaging for representative images was performed using an Andor Dragonfly spinning disk confocal microscope at 20x magnification with the ZL41 Cell sCMOS Zyla camera. Confocal imaging for data curation was performed using a Nikon Ti2 confocal microscope at 20x magnification with the DS-Qi2 CMOS camera. A ZEISS Axioscope 5 Fluorescent microscope was used to quantify gaps in GABAergic neurons.

Representative image processing was done in Fiji (image J) and dual channel intensity plots were developed using the multi-color-line-profile-plot macro. Image analysis for RAB-3 ::GFP and SNB-1 ::GFP intensity, puncta number, and area for each animal was performed using the puncta_analysis macro package in Fiji (Image J) as previously described (Hulsey-Vincent *et al.* 2023). Averages were collected from a minimum of 10 worms at each time point for both WT and TauTg. Statistical tests for these metrics were Student's T-test with Bonferroni correction.

To assess uniformity of distribution of RAB-3 and SNB-1 puncta, a 100µm ROI in the anterior dorsal cord was plotted for intensity along the X-axis using Fiji. ROIs with regions >5µm without a fluorescence peak (puncta) were classified as non-uniform (Fig 1O, P). A minimum of 10 animals were scored per genotype. Genotypes were compared using the Fisher Exact test. (* p < 0.05, ** p < 0.01).

Motor cord breaks were assessed at larval stage 4, day 1 and 6 of adulthood, the number of motor cord breaks in each animal was counted using fluorescence microscopy and averaged. A minimum of 4 sets of 10 were scored for each genetic background and age. Statistical comparison used was the student's t-test with Bonferroni correction (* p < 0.05, ** p < 0.01, *** p < 0.001).

&nbsp;

**RT-qPCR**

*C. elegans* were cultured as previously described and harvested at L4 and D6. RNA extraction was performed using a Zymo Quick-RNA™ MiniPrep. Reverse transcription of RNA samples was performed using LunaScript® RT SuperMix. cDNA samples were then amplified using iTaq® Universal SYBR Green Supermix in conjunction withtranscript specific primers (table 2). Amplification and real-time SYBR detection was performed using a Bio-Rad CFX Connect Real-Time PCR Detection System. A minimum of three Technical and biological replicates were performed and quantitated using the 2 ^-ΔΔCt^ method as previously described (Livak and Schmittgen 2001). Housekeeping genes ( *act-1* and *tba-1* ) were batch normalized using error propagation analysis. A t-test was then used to determine significance using the error propagation deviations.

## Reagents

***Strains and genetics*** *C. elegans* strains were maintained using standard procedures. All double mutants were constructed following standard procedures and were confirmed by associated phenotypes (GFP and or mCherry expression in neurons and pharynx).&nbsp;

**Table:** 

**Strain**	**Genotype**	**Source**	**Notes**
N2	wild-type, N2 bristol	CGC	WT control, each strain backcrossed 4X to N2
CK10	*bkIs10* [ *aex-3 p* ::tau(V337M 4R1N); *myo-2p* ::GFP] III	CGC	TauTg: pan-neuronal expression of HuTauV337M
NM2415	&nbsp; *jsIs682* [ *rab-3 p* ::GFP:: *rab-3* + *lin-15* (+)] III	CGC	assess RAB-3 in motor neurons
CZ13799	*juIs76* [ *unc-25p* ::GFP *+ lin-15* (+)] II	CGC	GFP label the GABAergic motor neurons
CZ333	*juIs1* [ *unc-25p* :: *snb-1* ::GFP + *lin-15* (+)] IV	CGC	assess SNB-1 in motor neurons
AMH11	*wpIs36* [ *unc-47p* ::mCherry] I *; sosIs5* [ *rab-3 p* ::Cerulean-Venus:: *lgg-1* + *unc-119* (+)]	CGC	mCherry label GABAergic motor neurons Outcrossed to removed *sosIs5*

&nbsp;

***Primers Used***

**Table:** 

**Primer name**	**Sequence**	**Template**
*rab-3* C18A3.6a.1 (fwrd)	CTTCTGCCTTCGTCTCTACTGTCG	cDNA
*rab-3* C18A3.6a.1 (rvs)	CTCCACGATAGTAGGCGGTGG	cDNA
*act-1* T04C12.6.1 (fwrd)	TCCTCCTCACTGAAGCCCCA	cDNA
*act-1* T04C12.6.1 (rvs)	GACGACTCCGGTGGTACGTC	cDNA
*aex-3* C02H7.3a.1 (fwrd)	CGAAGTCGTCCGCAATGCAC	cDNA
*aex-3* C02H7.3a.1 (rvs)	CGCCAACTTGCTCGGCACTC	cDNA
*tba-1* F26E4.8a.1 (fwrd)	TCAACACTGCCATCGCCGCC	cDNA
*tba-1* F26E4.8a.1 (rvs)	TCCAAGCGAGACCAGGCTTCAG	cDNA

**&nbsp;**

***Web resources & Software***

**Table:** 

**name**	**Version**	**link**
FIJI (ImageJ)	Version 1.54p	https://imagej.net/software/fiji/downloads
puncta_analysis macro	n/a	https://github.com/heinohv/puncta_analysis
MCLPP macro	n/a	https://github.com/KeesStraatman/Multi-color-line-profile-plot.git
Microsoft Excel	2604	https://www.microsoft.com/en-us/microsoft-365/excel
ApE	2.0.70	https://jorgensen.biology.utah.edu/wayned/ape/
WormBase	WS298	https://www.wormbase.org/
Adobe Illustrator	30.2	https://www.adobe.com/products/illustrator.html

***&nbsp;***
